# Investigation of the Diagnostic Value of Cerebrospinal Fluid and Serum sTREM-1 Levels in Neonatal Meningitis

**DOI:** 10.3390/children11081026

**Published:** 2024-08-22

**Authors:** Salih Çağrı Çakır, Bayram Ali Dorum, Hilal Özkan, Nilgün Köksal, Fatma Kocael, Ferah Budak, Mustafa Hacımustafaoğlu, Solmaz Çelebi, Muhammed Ali Kızmaz, Cansu Sivrikaya Yıldırım, Kevser Üstün Elmas

**Affiliations:** 1 Division of Neonatology, Department of Pediatrics, Faculty of Medicine, Bursa Uludag University, Bursa 16059, Turkey; hilalozkan@uludag.edu.tr (H.Ö.); nilgunk@uludag.edu.tr (N.K.); fatmakocael@uludag.edu.tr (F.K.); cansusivrikaya@uludag.edu.tr (C.S.Y.); kevserustun@uludag.edu.tr (K.Ü.E.); 2 Division of Neonatology, Department of Pediatrics, Bursa City Hospital, Bursa 16110, Turkey; bayramali.dorum@saglik.gov.tr; 3 Department of Immunology, Faculty of Medicine, Bursa Uludag University, Bursa 16059, Turkey; fbudak@uludag.edu.tr (F.B.); kizmaz.mali@gmail.com (M.A.K.); 4 Division of Pediatric Infectious Disease, Department of Pediatrics, Faculty of Medicine, Bursa Uludag University, Bursa 16059, Turkey; mkemal@uludag.edu.tr (M.H.); solmaz@uludag.edu.tr (S.Ç.)

**Keywords:** neonatal meningitis, neonatal sepsis, premature infant, soluble triggering receptor expressed on myeloid cells 1 (sTREM-1)

## Abstract

**Background:** The aim of this study is to investigate the diagnostic value of cerebrospinal fluid (CSF) and serum levels of the soluble form of triggering receptor-1 expressed on myeloid cells (sTREM-1) in neonatal meningitis. **Methods:** Serum sTREM-1 levels were measured in all neonatal sepsis patients at the start of antibiotic therapy and the 48th hour of treatment. At the beginning of antibiotic therapy, CSF samples were collected for sTREM-1 measurements. Control CSF samples were also collected from the patients with meningitis at the 48th hour of treatment. **Results:** A total of 77 preterm (50) and term (27) patients with neonatal sepsis were included in the study. There was no significant difference between the CSF sTREM-1 levels of patients with and without meningitis. The CSF sTREM-1 levels of preterm infants with meningitis decreased significantly after treatment (*p* = 0.038). Although the CSF/serum sTREM-1 ratios tended to increase in babies with meningitis, no significant difference was found between the groups. CSF/serum sTREM-1 ratios (mean ± SD) were 1.42 ± 0.91 and 1.14 ± 0.85 in preterm babies with and without meningitis and 1.15 ± 0.97 and 0.97 ± 0.55 in term babies with and without meningitis, respectively. **Conclusions:** Serum and CSF sTREM-1 levels increase in patients with neonatal sepsis. CSF s-TREM-1 levels decrease after treatment in preterm infants with meningitis.

## 1. Introduction

Meningitis is one of the most discussed and common life-threatening diseases in the neonatal period [1]. Its incidence in the neonatal period varies between 0.25 and 0.32 per 1000 live births [1]. Meningitis accompanies approximately 15–25% of neonatal sepsis cases [1,2]. Morbidity at a rate of 20–60% and mortality at a rate of 10–15% may develop due to neonatal meningitis [3]. Mortality is two times higher in premature babies [2]. To reduce the morbidity and mortality associated with neonatal meningitis, it is essential to have an early and definitive diagnosis and to administer antibiotics for an adequate duration and at appropriate doses. The clinical and laboratory findings of neonatal meningitis are similar to those of neonatal sepsis, and the diagnosis is made by cerebrospinal fluid (CSF) examination [1,2]. The gold standard diagnosis is demonstrating the agent in the culture [2]. However, just as there are cases with simultaneous positivity in blood and CSF cultures, positivity may not be detected in CSF cultures in one-third of cases [1]. In addition, positivity in blood cultures can be detected in only 0.5% of babies evaluated for neonatal sepsis [4]. Therefore, the CSF cell count and biochemical and microbiological evaluation of CSF have gained importance. The evaluation of CSF analysis in newborns has some limitations. Many patients receive antibiotic medications before CSF evaluation. In addition, the CSF white blood cell count of >20–30 cells/mm^3^ has a sensitivity and specificity of approximately 80% for meningitis [2].

Lumbar puncture is a challenging procedure for infants and requires experience in the neonatal period. Traumatic procedures are frequently encountered. In this case, it is necessary to consider the patient as having meningitis until the culture is concluded [1]. To diagnose meningitis, there is a need for reliable additional methods that can be examined both in serum and in CSF, apart from cell, biochemistry, and microbiological examinations in CSF.

The triggering receptors expressed on myeloid cells (TREMs) are transmembrane glycoproteins belonging to the immunoglobulin superfamily. These activating receptors are expressed on myeloid cell surfaces, such as monocytes, macrophages, and neutrophils, when encountering microorganisms [4,5,6,7]. Soluble TREM-1 (sTREM-1) is a 17 kDa soluble form formed by metalloproteinase cleavage and increases in serum and body fluids in cases of infection [5,6,7]. Studies conducted in newborns and other age groups have shown a close relationship between the presence, severity, and course of infections and sTREM-1 levels [7]. In addition, studies conducted in adults indicate that sTREM-1 may be a valuable and new marker for the definitive diagnosis of bacterial meningitis and for identifying patients with a high risk of adverse outcomes [6].

In this study, the diagnostic value of sTREM-1 levels in neonatal meningitis was investigated by studying sTREM-1 levels in the CSF and serum samples of patients with suspected neonatal meningitis.

## 2. Materials and Methods

### 2.1. Design

The study was conducted prospectively between 1 September 2019 and 1 September 2022 in a tertiary neonatal intensive care unit. The study began after the approval of the Bursa Uludag University Ethics Committee (approval number: 2021-4/26 and approval date: 5 March 2019). The study was supported by the Bursa Uludag University Scientific Research Fund (No: TGA-2021-370). Informed consent was obtained from the families.

### 2.2. Population

Patients who were considered to have neonatal sepsis and were diagnosed with sepsis by the European Medicines Agency were included in the study [2]. CSF and blood cultures were obtained from all patients. Patients with positive CSF or blood cultures were considered to have culture-proven sepsis. In the CSF sample, white blood cell count > 20–30 cells/mm^3^, protein amount > 150 mg/dL in preterm infants, protein amount > 100 mg/dL in term infants, glucose level < 20 mg/dL in preterm infants, and glucose level < 30 mg/dL in term infants or lower than 70% concurrent blood glucose value were accepted as meningitis evidence [2]. Meeting one of these criteria was considered sufficient for the diagnosis of meningitis.

Serum and CSF samples were taken from the patients at the time of diagnosis (day 0) and at the 48th hour of treatment (day 2) for sTREM-1 level measurement. Patients for whom consent could not be obtained, patients who could not have a lumbar puncture, or those whose sample could not be obtained were excluded from the study.

### 2.3. Analysis of Samples

The blood taken for the sTREM-1 level was kept at room temperature for 10–20 min, and then the serum was separated by centrifugation at 2000–3000 revolutions per minute for 20 min. The CSF samples were similarly separated after centrifugation for 20 min. Separated sera and CSF samples were stored at −80 degrees. sTREM-1 levels were studied using the enzyme-linked immunosorbent assay method (Bioassay Technology Laboratory (Shanghai, China), Cat. No E0310Hu, sensitivity: 2.53 pg/mL, standard range: 5–2000 pg/mL).

### 2.4. Statistics

Statistical analyses were performed using the IBM SPSS 28.0 statistical program. Descriptive data are given as mean (standard deviation), median (interquartile range or minimum − maximum), frequency, and percentage. The distribution characteristics of continuous variables between groups were evaluated with the Shapiro–Wilk test. Groups were compared with Student’s t-test for normally distributed data and the Mann–Whitney U test for data not normally distributed. Categorical variables in the groups were compared using the chi-square test. A paired *t*-test or Wilcoxon test was used to compare the first and subsequent measurements in the study group. A *p*-value of <0.05 obtained by statistical analysis was considered significant. Receiver operating characteristic (ROC) curve analysis was performed using a software program (MedCalc^®^ version 22.032). Then, sensitivity, specificity, and positive and negative likelihood ratios were calculated with respect to determined cut-off values. The sample size was calculated with the G*Power 3.1.9.6 program.

## 3. Results

**Figure 1:**
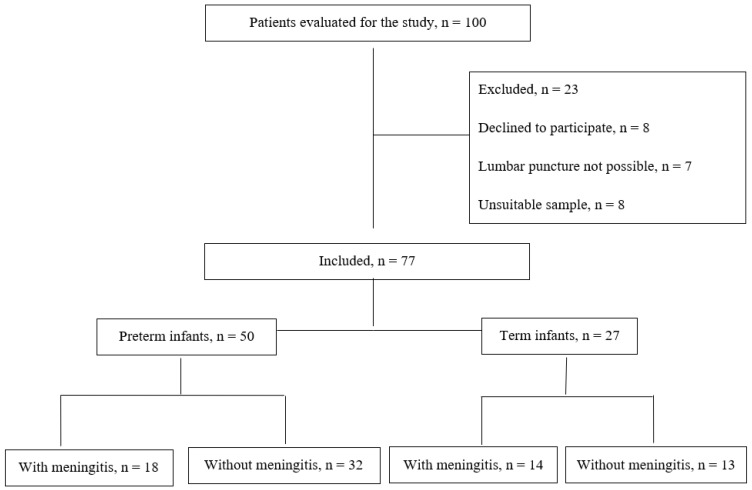
The flow chart of the patients included in the study.

The flow chart of the patients included in the study is shown in Figure 1. Fifty patients were premature (mean ± SD; 31.7 ± 3.1 weeks), and twenty-seven were term (mean ± SD; 38.2 ± 0.9 weeks). Fourteen (52%) term babies and eighteen (36%) preterm babies were diagnosed with meningitis by CSF examination. Fourteen (77%) preterm neonates’ and thirteen (93%) term neonates’ CSF protein amount diagnosed with meningitis fulfilled the diagnostic criteria. Fifteen (83%) preterm neonates’ and four (29%) term neonates’ CSF white blood cell count diagnosed with meningitis fulfilled the diagnostic criteria. Sixteen (89%) preterm neonates’ and six (43%) term neonates’ CSF glucose levels diagnosed with meningitis fulfilled diagnostic criteria.

The demographic and clinical data of the infants included in the study are given in Table 1.

The serum and CSF sTREM-1 levels of the babies at the time of sepsis diagnosis are given in Table 2.

When the serum and CSF sTREM-1 levels of term and preterm babies diagnosed with meningitis were compared before the treatment and at the 48th hour of the treatment, it was observed that the CSF sTREM-1 levels and the CSF/serum sTREM-1 levels decreased after the treatment. It was observed that the decrease in CSF sTREM-1 levels in premature babies reached statistical significance (Table 3).

**Figure 2:**
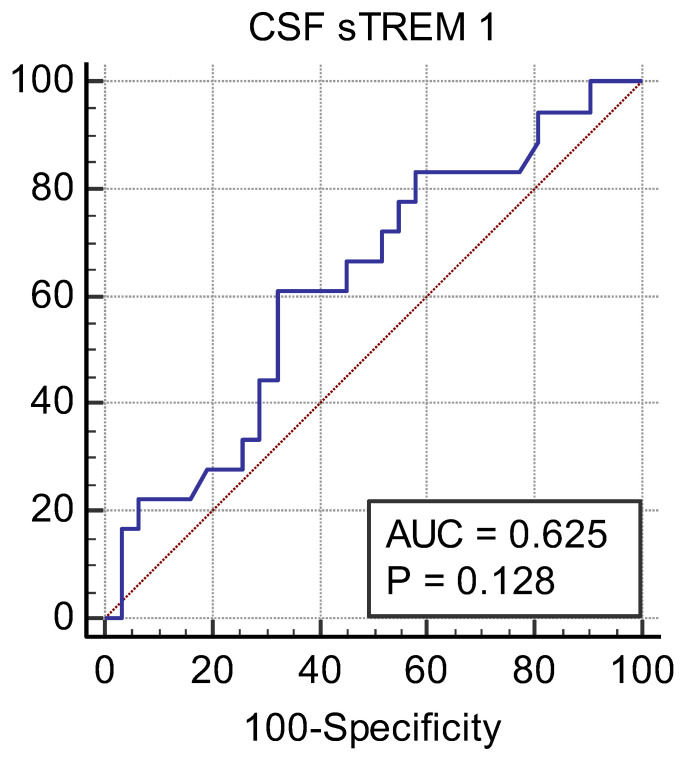
The ROC analysis for preterm neonates.

**Figure 3:**
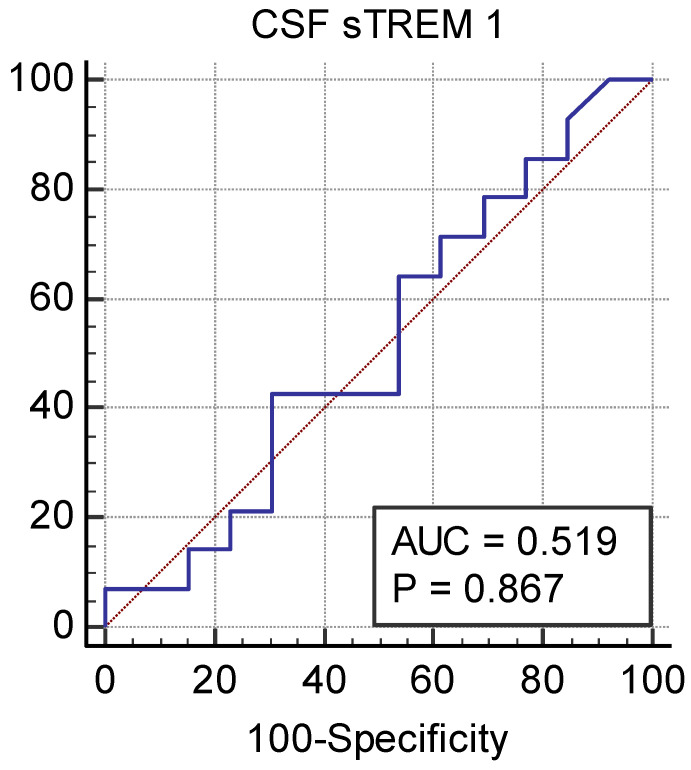
The ROC analysis for term neonates.

The sensitivity, specificity, area under the curve (AUC), and cut-off level of CSF sTREM-1 were calculated. The ROC analysis of the patients is shown in Figure 2 and Figure 3. The AUC was 0.625 [95% confidence interval (CI): 0.476–0.760, *p* = 0.128] for CSF sTREM-1 in preterm neonates. In preterm neonates, for cut-off point 306.3 pg/mL of the CSF sTREM-1, the sensitivity, specificity, negative likelihood ratio, and positive likelihood ratio were 61.11 (95% CI: 35.7–82.7), 67.74 (95% CI: 48.6–83.3), 0.57 (95% CI: 0.31–1.08), and 1.89 (95% CI: 1.01–3.55), respectively. The AUC was 0.517 (95% CI: 0.320 to 0.714, *p* = 0.867) for CSF sTREM-1 in term neonates. In term neonates, for cut-off point 305.9 pg/mL of the CSF sTREM-1, the sensitivity, specificity, negative likelihood ratio, and positive likelihood ratio were 42.86 (95% CI: 17.7–71.1), 69.23 (95% CI: 38.6–90.9), 0.83 (95% CI: 0.46–1.48), and 1.39 (95% CI: 0.50–3.84), respectively.

The causative agent was detected in the blood cultures of two term patients and six premature babies in equal numbers in the groups with and without meningitis. There were no patients in whom the causative agent could be isolated in CSF cultures. There was no infection-related mortality in the study group.

## 4. Discussion

In this study, CSF sTREM-1 levels were studied for the first time, as far as we know, in patients with neonatal sepsis and meningitis. In addition, the course of both serum and CSF sTREM-1 levels was revealed in the follow-ups of the patients. CSF sTREM-1 levels were similar in all patients. However, in this study, it was shown that CSF sTREM-1 levels decreased after the initiation of treatment in premature babies with meningitis. No significant difference was found in the sTREM-1 levels of newborns diagnosed with clinical sepsis, which can be used in the diagnosis of patients with meningitis.

There is no normal or diagnostic serum or CSF sTREM-1 level in term or preterm infants. Previous studies have reported that sTREM-1 levels are significantly lower in preterm and term babies without signs of infection compared to those with infection [7,8,9,10,11]. sTREM-1 levels were found to be 29.1 pg/mL (14.55–103.93) in premature babies without signs of infection [11]. Although a negative correlation was found between sTREM-1 levels and only birth weight in these babies, no statistically significant correlation was found with gestational age, maternal age, gender, mode of delivery, patent ductus arteriosus, intrauterine growth retardation, or premature rupture of membranes [11]. In the healthy control group in another study, sTREM-1 levels were found in preterm babies at 207.5 ± 62 pg/mL, and at term, they were found to be 126.5 ± 29 pg/mL [10]. In this study, for ethical reasons, a healthy control group in which CSF and serum sTREM-1 levels were measured was not formed. In our study, term and preterm babies were evaluated separately.

Although studies have shown that sTREM-1 levels can be used as a useful biomarker for the diagnosis of neonatal sepsis, the cut-off values used vary between 77.5 and 1707.35 pg/mL [7]. In a study examining different biomarkers in babies older than 34 weeks of gestation for the diagnosis of early neonatal sepsis, the optimal cut-off value for sTREM-1 was calculated as 1250 pg/mL [12]. An insignificant increase in sTREM-1 levels was found in septic infants compared to uninfected infants (1436 vs. 1234 pg/mL, *p* = 0.055) [12]. Saldir et al. [13] found that sTREM-1 and interleukin-6 levels were good markers for the diagnosis of late neonatal sepsis. They reported that sTREM-1 levels were higher in babies diagnosed with sepsis than in the control group: sTREM-1 (pg/mL) median 985, interquartile range (IQR) 576–1400 and sTREM-1 (pg/mL) median 73, IQR 60–124.5, respectively, with *p* < 0.001 [13]. They found the sTREM-1 cut-off value to be 450 (pg/mL) for the diagnosis of late sepsis [13]. In another study, an sTREM-1 value of 310 pg/mL was recommended as a cut-off value for the diagnosis of neonatal sepsis [10].

It has been shown that blood sTREM-1 may be a good marker for the diagnosis of ventilator-associated pneumonia (VAP) in newborns [14]. It has been reported that 72nd-hour-blood sTREM-1 levels are significantly higher in patients with VAP than in those without it (14). The optimal cut-off value for the diagnosis of VAP was 165.05 pg/mL [14]. Alkan et al. [8] found that urine sTREM-1 levels (pg/mL) were significantly higher in preterm infants with late sepsis and positive blood cultures. They calculated the urinary sTREM-1 cut-off value as 78.5 pg/mL for culture positivity in these patients [8]. This finding in sepsis is important in terms of showing the increase in sTREM-1 in body fluids other than blood. All of the patients in our study were diagnosed with sepsis, and both serum and CSF sTREM-1 levels increased.

Chen et al. [15] reported that sTREM-1 levels were significantly higher in infants younger than three months of age with culture-proven severe bacterial infection (mean ± SD, 324.6 ± 546.3 and 7.7 ± 16.4; *p* = 0.0109). However, a clear relationship between the increase in sTREM-1 levels in neonatal sepsis and the severity of the infection has not been demonstrated in the literature. In a study involving both term and preterm infants, the sTREM-1 levels of culture-positive and culture-negative neonatal sepsis patients were found to be similar (4). Sarafidis et al. [9] also reported that sTREM-1 levels were similar in term and preterm babies with culture-positive and negative neonatal sepsis, and they determined the cut-off level of sTREM-1 to be 143.35 pg/mL. Adly et al. [10] found sTREM-1 blood levels to be similar in newborns with sepsis with and without culture growth but significantly higher than in the control group. Tunc et al. [16] reported that there was no significant difference in sTREM-1 levels in the control group, proven, and probable sepsis infants. TREM-1 expression has been shown in myeloid cells of both premature and term newborns [11,17]. In this study’s patients, the diagnosis of sepsis was made according to clinical and laboratory findings, and the causative agent was detected in the blood cultures of only two term and six premature babies.

There are no data on CSF sTREM-1 levels in newborns in the literature, and studies conducted in adults have shown that CSF sTREM-1 levels can be beneficial in the diagnosis of bacterial meningitis. In a study conducted in adults, CSF sTREM-1 levels were found in those with bacterial meningitis: 82 pg/mL (0–988); in those with viral meningitis: 0 pg/mL, (0–48); and in the control group: 0 pg/mL (0–36) [5]. The cut-off value of sTREM-1 for the diagnosis of bacterial meningitis was reported to be 20 pg/mL [5]. Our study did not detect the CSF sTREM-1 level that has diagnostic value for meningitis. ROC curve analyses showed that CSF sTREM-1 resulted in a nonsignificant AUC for identifying neonatal meningitis.

In another study conducted in adults, the CSF sTREM-1 levels of patients with bacterial meningitis were found to be higher than those of non-bacterial meningitis cases, and the cut-off value was reported as 11,515 pg/mL [18]. Bishara et al. [19] also found significantly higher CSF sTREM-1 levels in bacterial meningitis than in aseptic meningitis. However, CSF sTREM-1 levels and blood sTREM-1 levels were not evaluated in these studies. It has been stated that sTREM-1 detected in CSF may be associated with leukocytes in the CSF area or with the passage of sTREM-1 in the bloodstream [5]. In our study, similar levels of CSF sTREM-1 were detected in newborn infants without meningitis as in newborn infants with meningitis, and it was thought that this situation might be related to the increase in sTREM-1 due to sepsis. Serum sTREM-1 levels were found to be significantly higher in preterm babies without meningitis. However, the increasing trend in CSF/serum sTREM-1 ratios of preterm and term infants with meningitis suggests that sTREM-1 levels show a greater increase in CSF in relation to meningitis. In addition, a decrease in the control CSF sTREM-1 ratios after treatment in infants with meningitis, which reached a significant level in preterm infants and did not reach a significant level in term infants, and a decrease in CSF/serum sTREM-1 ratios, which did not reach a significant level in both patient groups, strongly suggest a relationship between CSF sTREM-1 levels and meningitis. In fact, Saldir et al. [13] reported that the sTREM-1 levels of patients with neonatal sepsis decreased significantly after the initiation of treatment. In another study, a significant decrease in sTREM-1 levels was reported after treatment in patients with neonatal sepsis with and without culture growth [10]. Although the literature data show that sTREM-1 levels can be used in the diagnosis and follow-up of neonatal sepsis, the cut-off levels found in studies vary widely because they are limited to study populations, and a recommendation level to be used in all patients cannot be determined.

The small number of culture-positive cases and the absence of a control group consisting of preterm and term infants without signs of sepsis were considered limitations of the study. The sample size was calculated separately for premature and term neonates based on our CSF sTREM-1 results with the G*Power 3.1.9.6 program for future studies. The sample size was determined as 186 and 394 for preterm and term neonates, respectively, with the G Power program, by taking α = 0.05, power (1 − β) = 0.80, and effect size 0.41 (preterm neonates) and 0.28 (term neonates) at a confidence level of 95%.

## 5. Conclusions

The findings of this study show that serum and CSF sTREM-1 levels increase in patients with neonatal sepsis, CSF/serum sTREM-1 ratios tend to increase in cases of meningitis, and CSF sTREM-1 levels and CSF/serum sTREM-1 ratios tend to decrease after treatment in neonatal meningitis. Therefore, prospective cohort studies examining more patients with culture evidence of meningitis are needed to determine the diagnostic and prognostic role of sTREM-1 levels in neonatal meningitis.

## Figures and Tables

**Table 1:** Demographic and clinical data of the infants.

	Term Neonates, *n* = 27		*p*	Preterm Neonates, *n* = 50		*p*
	With meningitis, *n* = 14	Without meningitis, *n* = 13		With meningitis, *n* = 18	Without meningitis, *n* = 32	
Gestational age (week), median (min-max)	38.2 (37–40)	38.3 (37–39)	0.903 ^a^	31.5 (26.3–36.5)	31.3 (26.5–36.6)	0.816 ^a^
Birth weight (gr), median (min-max)	3260 (2470–4180)	2970 (2400–3800)	0.234 ^a^	1587 (780–3100)	1625 (860–3300)	0.657 ^a^
Gender (*n*, Female/male)	8/6	7/6	0.863 ^b^	6/12	18/14	0.119 ^b^
Cesarean section (*n*)	7	7	0.842 ^b^	18	28	0.283 ^b^
Maternal age (year), median (min-max)	28 (23–34)	31 (24–42)	0.381 ^a^	29 (22–40)	30 (19–40)	0.562 ^a^
Apgar 1. minute, median (min-max)	8 (3–9)	8 (1–9)	0.612 ^a^	7 (0–9)	7 (2–9)	0.947 ^a^
Apgar 5. minute, median (min-max)	9 (7–10)	9 (1–10)	0.489 ^a^	9 (2–9)	8 (6–10)	0.855 ^a^
Postnatal age (day), median (min-max)	4 (2–25)	3 (2–25)	0.673 ^a^	5 (2–44)	4 (1–30)	0.820 ^a^
Early/late neonatal sepsis (*n*)	3/11	4/9	0.580 ^b^	3/15	5/27	0.923 ^b^

^a^; Mann–Whitney U test, ^b^; chi-square test.

**Table 2:** The serum and CSF sTREM-1 levels of the babies at the time of sepsis diagnosis.

	Term Neonates, *n* = 27		*p*	Preterm Neonates, *n* = 50		*p*
	With meningitis, *n* = 14	Without meningitis, *n* = 13		With meningitis, *n* = 18	Without meningitis, *n* = 32	
Serum sTREM1 (pg/mL), median (IQR)	354.1 (187.5–545.5)	254.1 (211.1–538.8)	0.951 ^a^	252.2 (145.2–308.6)	296.7 (234.7–447.7)	0.038 ^a^
CSF sTREM1 (pg/mL), median (IQR)	252.8 (209.6–384.2)	262.4 (188.7–406.4)	0.865 ^a^	290.4 (218.6–360.6)	339.3 (240.8–390.8)	0.147 ^a^
CSF/serum sTREM-1, mean ± SD	1.15 ± 0.97	0.97 ± 0.55	0.583 ^b^	1.42 ± 0.91	1.14 ± 0.85	0.294 ^b^

^a^; Mann–Whitney U test, ^b^; *t*-test, IQR; interquartile range, CSF; cerebrospinal fluid, SD; standard deviation.

**Table 3:** The serum and CSF sTREM-1 levels of neonates with meningitis.

	Term Neonates, *n* = 14			Preterm Neonates, *n* = 18		
sTREM1 (pg/mL)	Serum	CSF	CSF/serum sTREM-1	Serum	CSF	CSF/serum sTREM-1
Day 0, median, (IQR)	354.1 (187.5–545.5)	252.8 (209.6–384.2)	0.84 (0.35–1.93)	252.2 (145.2–308.6)	290.4 (218.6–360.6)	1.17 (0.80–1.79)
Day 2, median (IQR)	281.5 (228.0–403.6)	238.8 (203.1–410.6)	0.72 (0.65–1.44)	252.7 (195.7–307.1)	226.0 (201.8–357.3)	0.84 (0.48–1.09)
P	0.398	0.929	0.866	0.309	0.038	0.086

P; Wilcoxon signed ranks test, IQR; interquartile range, CSF; cerebrospinal fluid.

## Data Availability

Necessary data are included within the manuscript. Explicit consent for the open sharing of the data was not obtained. The data are not publicly available due to privacy.
